# Phubbing in pregnancy as a predictor of postpartum depressive symptoms: preliminary results

**DOI:** 10.1186/s40359-026-04991-8

**Published:** 2026-07-07

**Authors:** Małgorzata Sobol, Elisa Mancinelli, Alessandra Schiavo, Nicola Cellini, Giovanna Mioni, Agata Błachnio, Silvia Salcuni

**Affiliations:** 1 Uniwersytet Warszawski, Wydział Psychologii, ul. Banacha 2D, Warszawa, 02-097 Polska; 2 Universita Degli Studi Di Padova, Via VIII Febbraio, 2, Padua, 35122 Italia; 3 Schiavo’s Studio, Piazza Alcide De Gasperi 41, Padua, 35131 Italia; 4 Katolicki Uniwersytet Lubelski Jana Pawła II, Al. Racławickie 14, Lublin, 20-950 Polska

**Keywords:** Phubbing, Partner, Pregnancy, Postpartum depression

## Abstract

Partner behavior toward pregnant women plays a significant role in maternal mental health. One of the common behaviors that negatively affects the quality of communication is phubbing. Phubbing is defined as ignoring a partner during face to face interaction in favor of a mobile phone. The present study examined the association between partner phubbing during pregnancy and postpartum depression. Thirty expectant couples were recruited from a gynecological outpatient clinic in Italy and assessed longitudinally during the first trimester, third trimester, and within three months postpartum. Women completed the Edinburgh Postnatal Depression Scale (EPDS) and seven-day web-based observational diaries assessing perceived partner phubbing at each time point, while male partners completed the Phubbing Scale. Results indicated that women’s perceived experiences of partner phubbing during the third trimester statistically predicted the severity of postpartum depressive symptoms. In contrast, partners’ self-reported phubbing frequency was not associated with women’s postpartum depression. These findings underscore the importance of psychoeducation for couples during pregnancy regarding phubbing and its potential negative consequences, and identify the third trimester as a particularly sensitive period for partner behaviors related to attention and respect.

**Supplementary Information:**

The online version contains supplementary material available at https://doi.org/10.1186/s40359-026-04991-8.

## Introduction

The perinatal period, covering pregnancy and the early months postpartum, involves significant psychological and physiological changes that increase women’s vulnerability to mental health disorders [17]. Postpartum depression is among the most common, with a global prevalence of 17.22% [29]. It is characterized by affective, cognitive, and behavioral symptoms, including depressed mood, anhedonia, guilt, sleep and appetite disturbances, and reduced libido, as well as anxiety, irritability, emotional lability, social withdrawal, and, in severe cases, suicidal ideation or thoughts of harming the child [3]. The etiology of postpartum depression is multifactorial, involving interacting psychosocial, demographic, and biological factors [1, 5]. Identifying predictors of postpartum depression during pregnancy is crucial for prevention, and routine prenatal visits provide key opportunities to detect risk and implement early interventions [17]. One of the factors significantly associated with symptoms of postpartum depression in a woman is her partner’s behavior toward her [12]. Partner emotional support, which includes the expression of positive emotions, sensitivity to difficulties, and responsiveness to a woman’s needs, plays a crucial role in women’s psychological well being during the perinatal period [20, 24, 26]. Maintaining relationship quality at this time depends on effective, attentive communication, which is increasingly disrupted by mobile phone use [8, 9].

Research shows that phubbing negatively affects close relationships. It is typically conceptualized as a form of interpersonal disengagement during face to face interaction in which attention is redirected to mobile phone use instead of responding to the partner [16, 28]. This can be explained by the Interpersonal Process Model of Intimacy [23], which views intimacy as a dynamic cycle of self-disclosure and partner responsiveness. When responsiveness is replaced by phone distraction, this cycle is interrupted, reducing perceived support, weakening emotional closeness, and ultimately lowering relationship quality.

Phubbing is consistently linked to poorer romantic relationship functioning and lower relationship satisfaction [2, 22]. It has also been shown to predict depressive symptoms in late pregnancy, with low marital satisfaction partially mediating this association [31]. Additionally, Frackowiak et al. [10] found that daily experiences of partner phubbing are associated with lower perceived responsiveness and more negative evaluations of the partner’s behavior. To the best of our knowledge, no previous studies have investigated the relationship between partner phubbing during pregnancy and postpartum depression. The present study therefore aims to examine whether partner phubbing in the first and third trimesters of pregnancy predicts the severity of postpartum depressive symptoms in women. The proposed model is grounded in the construct of phubbing [16] and the Interpersonal Process Model of Intimacy [23].

During pregnancy, women tend to exhibit increased sensitivity to partner behaviors and a heightened need for emotional support [14, 15]. Perceived support and understanding are largely shaped by the quality of dyadic communication, which may be compromised by phubbing [16]. Such behavior has been associated with negative emotional reactions, including feelings of neglect and devaluation [2, 22]. Given robust evidence linking maternal mental health during pregnancy with postpartum psychological outcomes, we hypothesize that higher levels of partner phubbing during pregnancy will be associated with greater severity of postpartum depressive symptoms.

## Methods

### Participants

A total of 60 participants (*n* = 30 women; *n* = 30 men) were recruited from a gynecological outpatient clinic in Padua. All participants were Italian citizens. Women and men aged over 17 years were eligible for inclusion. Exclusion criteria included the presence of severe mental disorders and multiple pregnancy. All participants provided informed consent to take part in the study on a voluntary basis.

### Procedure

The study employed a longitudinal design and consisted of three stages: during the first gynecological visit in the first trimester of pregnancy, in the third trimester of pregnancy, and within the first three months postpartum (21–92 days after the childbirth, M = 60.90 days, SD = 22.44). At each stage, women underwent a medical examination and received links to online questionnaires, as well as access to a mobile phone application. Male partners received a link to an online questionnaire. The frequency of men’s phubbing behaviors was assessed from both the men’s perspective (using a self-report measure) and the women’s perspective (using observation diaries). Women’s depressive symptoms were measured using a self-report instrument completed by the women.

### Measures

Sociodemographic and medical data were collected using a structured questionnaire. The sociodemographic section included items assessing age, place of residence, occupation, level of education, income, and history of mental disorders. Women were additionally asked about the presence of chronic illnesses and the regular use of medication. Medical data comprised information obtained from routine gynecological examinations, including fetal development, the presence of symptoms indicating risk of miscarriage or preterm birth, and comorbid medical conditions.

Perinatal depressive symptoms were assessed using the Edinburgh Postnatal Depression Scale (EPDS), in the Italian adaptation by Benvenuti et al. [4], which consists of 10 items rated on a 4-point Likert scale (e.g., “I have been able to laugh and see the funny side of things: as much as I always could,not quite so much now; definitely not so much now; not at all”). In the present study, the EPDS demonstrated satisfactory internal consistency (Cronbach’s alpha = 0.83 in the first trimester, 0.85 in the third trimester, and 0.80 postpartum).

Women’s perceived experiences of partner phubbing were assessed using a smartphone-based mobile application, developed specifically for the purposes of the present study. The application included two items: “How many times today did you feel ignored because your partner was using their phone while you were together?” and “How many times today did you feel uncomfortable because your partner was using their phone while you were together?” Responses to each item were recorded on a 5-point Likert scale. Women completed the application daily for seven consecutive days at each of the three study stages. The total score was calculated as the sum of both items. Cronbach’s alpha coefficients were 0.85 in the first trimester, 0.88 in the third trimester, and 0.83 postpartum.

Men’s phubbing behaviors were assessed using the Phubbing Scale [16], in the Italian adaptation by Guazzini et al. [13]. This instrument consists of 10 items (e.g., “My eyes start wandering to my phone when I am with other people”, “People complain that I deal with my mobile phone”), which measure the extent to which mobile phone use interferes with face-to-face interactions, as well as the persistent urge and desire to use a mobile device. Responses were given on a 5-point Likert scale. In the present study, internal consistency (Cronbach’s alpha) was 0.70 in the first trimester, 0.75 in the third trimester, and 0.71 postpartum. The self-report method used to measure phubbing behaviors in men was chosen because diary-based assessments could have led them to intentionally limit such behaviors, as increased awareness might prompt avoidance. In contrast, a retrospective measure did not carry this risk.

### Data analysis

Participants with missing values on key variables were excluded from the analyses via listwise deletion. Descriptive statistics and correlation coefficients were computed using IBM SPSS Statistics version 27. A hierarchical regression analysis was conducted to examine whether phubbing during pregnancy is a statistical predictor of postpartum depressive symptoms. Moreover, to estimate the prospective associations of perceived partner phubbing in the first and third trimesters of pregnancy with postpartum depressive symptoms, cross-lagged panel analyses were conducted using the *lavaan* package in the R statistical environment. Cross-lagged analysis is a statistical technique commonly applied in longitudinal research to assess the direction and strength of associations between variables across multiple time points. This method allows for the examination of whether variability in one variable can be statistically predicted by earlier levels of another variable. Due to the small sample size, we did not include additional control variables in the cross-lagged analysis. Model fit indices were not reported for the two-wave cross-lagged panel model because the model was just-identified (df = 0). Just-identified models fully reproduce the observed covariance matrix by estimating all possible paths, which precludes the estimation of global model fit indices.

## Results

### Participant characteristics

A total of 58 individuals completed the first stage of the study (30 women aged 23–39 years; M = 33.53, SD = 3.57, and 28 men aged 29–48 years; M = 37.00, SD = 4.68). Two men did not complete the online questionnaire and did not provide reasons for non-participation. The second and third stages of the study were completed by 57 participants (29 women aged 23–39 years; M = 33.59, SD = 3.62, and 27 men aged 29–48 years; M = 37.11, SD = 4.74); one woman experienced a miscarriage during the first trimester of pregnancy. Post hoc power analysis was conducted using G*Power. Assuming a one-tailed test, the statistical power to detect a medium effect size in Pearson’s correlation analyses (*r* = 0.30) was approximately 0.60 (α = 0.05, one-tailed). For multiple regression analysis including two predictors, the estimated power to detect a medium effect size (f^2^ = 0.15), was approximately 0.55 (α = 0.05). For cross-lagged effects of similar magnitude, the statistical power was estimated to range between 0.55 and 0.65, indicating moderate sensitivity for detecting medium-sized longitudinal associations.

Sociodemographic and clinical characteristics of the sample (*N* = 57) are presented in Table 1. Among the surveyed women, 65% had secondary education and 35% had higher education. Among the men, 75% had secondary education and 25% had higher education. 47% of the couples were married, while 53% were in informal relationships. None of the women were taking medication on a regular basis, and three women reported having a chronic illness. The vast majority of the couples lived in a medium town (83%); two women lived in a large city, and three women lived in rural areas. Most couples reported having a very good financial situation (61%), while 39% described their financial situation as good. 20% of women obtained scores above 12 on the EPDS.

**Table 1:** Demographic and clinical characteristics of the sample

Women (*n* = 29)—Highest level of education	Elementary school 0 (0%)	19 (65%)	10 (35%)
Men (*n* = 27)—Highest level of education	0 (0%)	21 (75%)	6 (255)
Women (*n* = 29)—The presence of a chronic disease	yes 3 (7%)	no 26 (93%)	
Women (*n* = 29)—Long-term medication use	yes 0 (0%)	no 29 (100%)	
Women’s marital status (*n* = 29)	Single/Divorced 0 (0%)	Partnered 16 (53%)	Married 13 (47%)
Women’s place of residence (*n* = 29)	Village 3 (10%)	Medium town 24 (83%)	Big city 2 (7%)
Women’s financial situation (*n* = 29)	Medium 0 (0%)	Good 12 (39%)	Very good 17 (61%)

### Correlations among variables

Descriptive statistics and correlation coefficients among the study variables are presented in Table 2. Perceived partner phubbing in the third trimester was positively correlated with the severity of postpartum depressive symptoms. Perceived partner phubbing in the first trimester was associated with depressive symptom severity in the third trimester. Similarly, perceived partner phubbing postpartum was positively correlated with the intensity of postpartum depressive symptoms. Notably, men’s self-reported phubbing behaviors were not correlated with women’s perceptions of being phubbed by their partner. Men’s self-reported phubbing behaviors were also not associated with women’s depressive symptoms.

**Table 2:** Correlations amongst studied variables

	1	2	3	4	5	6	7	8	9	10	11	12	13	14	15
1. women age	-														
2. women education	.15	-													
3. men age	.37	.02	-												
4. men education	-.17	.72***	-.19	-											
5. financial situation	.18	.10	.12	.14	-										
6. place of residence	.09	.03	-.02	.06	.23	-									
7. women perceived phubbing I	.16	-.13	.11	-.31	.19	.22	-								
8. women perceived phubbing III	.17	-.26	.21	-.27	.15	.05	.15	-							
9. women perceived phubbing PP	.12	-.21	-.05	-.02	.11	-.22	.02	.64***	-						
10. men PS I	.11	.20	.02	.21	.14	-.09	.12	-.01	.15	-					
11. men PS III	.09	.12	.08	.17	.19	.11	.03	.12	.20	.44*	-				
12. men PS PP	.15	.19	.11	.15	.23	.19	.11	.18	.14	.39	.45*	-			
13. women EPDS 1	.03	.23	-.01	.13	.21	-.08	.05	.12	.25	.23	.21	.20	-		
14. women EPDS III	.09	.03	.25	-.26	.13	.03	.57**	.37	.27	.14	.18	.09	.49**	-	
15. women EPDS PP	.14	-.22	.07	-.22	.12	-.02	.10	.67***	.45*	.17	.15	.12	.55**	.51**	-
M	33.53	-	37	-	-	-	2.31	2.46	2.12	21.20	19.90	21.85	6.97	8.14	7.85
SD	3.57	-	4.68	-	-	-	.92	.74	.75	4.92	4.05	4.42	4.81	4.32	4.95

*EPDS* Edinburgh Postnatal Depression Scale, *PS* Phubbing Scale, *I* measurement in the first trimester, *III* measurement in the third trimester, *PP* measurement after delivery

^*^ *p* <.05

^**^ *p* <.01

^***^ *p* <.001

### Association between perceived partner phubbing and women’s postpartum depressive symptoms: hierarchical regression and cross-lagged analysis

To examine the association between phubbing during pregnancy and postpartum depressive symptoms, a hierarchical regression analysis was performed (Table 3). Depressive symptoms reported during the first trimester of pregnancy were included in the model as a control variable. Neither the presence of a chronic disease, marital status, age of either the woman or the man, educational level of both partners, the couple’s financial status, place of residence, nor depressive symptoms in the woman during the third trimester of pregnancy were significantly associated with postpartum depressive symptoms (Supplementary materials); therefore, they were not included as control variables in the analyses. As the independent variable, women’s perception of phubbing during the third trimester of pregnancy was included, as this variable was significantly correlated with postpartum depressive symptoms. Statistical predictors of postpartum depressive symptoms were perceived phubbing by the male partner in the third trimester of pregnancy and depressive symptoms in the first trimester of pregnancy. The more frequently women felt phubbed by their partners in the third trimester and the more severe the depressive symptoms they experienced in the first trimester, the greater the severity of postpartum depressive symptoms after childbirth.

**Table 3:** Hierarchical multiple regression results for prenatal predictors of postpartum depressive symptoms

Variables	t	β	R^2^	∆R^2^	∆F
Model 1			.23		
Step 1				.26	7.41*
women EPDS 1	2.72*	.51			
Model 2			.61		
Step 1					
women EPDS 1	3.33**	.45			
Step 2				.38	21.29***
women perceived phubbing III	4.61***	.62			

*EPDS* Edinburgh Postnatal Depression Scale, *I* measurement in the first trimester, *III* measurement in the third trimester

^*^ *p* <.05

*p* <.01

^***^ *p* <.001

**Fig. 1:**
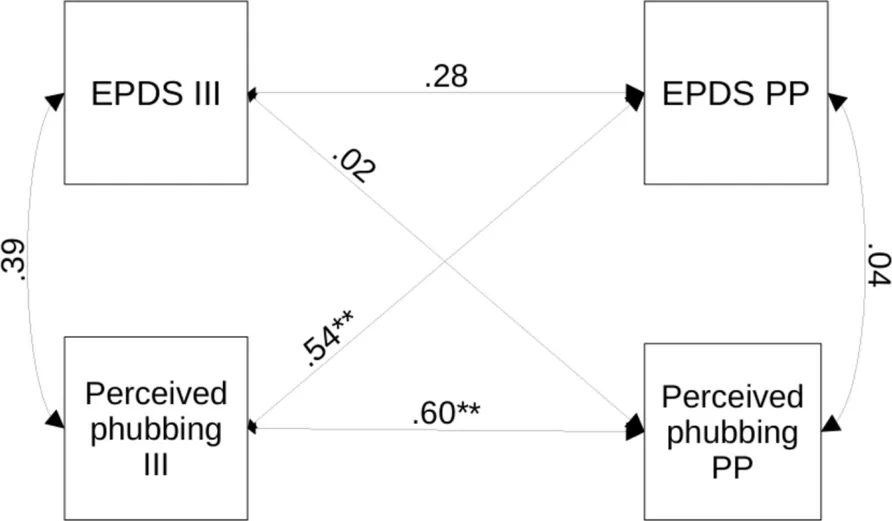
Relationships between women's perceived phubbing by their partner in the third trimester (Perceived Phubbing III) and postpartum (Perceived Phubbing PP), and depressive symptoms in the third trimester (EPDS III) and postpartum (EPDS PP)

Next, cross-lagged panel analysis was conducted to examine the associations between perceived partner phubbing in the third trimester of pregnancy and postpartum depressive symptoms. The results of the cross-lagged analyses indicated that perceived partner phubbing during the third trimester of pregnancy was a statistical predictor of the severity of postpartum depressive symptoms (see Fig. 1, Supplementary materials).

## Discussion

The present study aimed to examine the prospective association of partner phubbing during pregnancy with the severity of postpartum depressive symptoms in women. The proposed hypothesis was partially supported. Women’s perceived experiences of partner phubbing during the third trimester of pregnancy were associated with greater severity of postpartum depressive symptoms. However, there was no association between women’s perceived experiences of partner phubbing during the first trimester of pregnancy and postpartum depressive symptoms. Similarly, the frequency of phubbing by the partner, as reported from the man’s perspective, was not related to the woman’s postpartum depressive symptoms.

A significant relationship between women’s perceived experiences of partner phubbing and postpartum depressive symptoms is consistent with previous research highlighting the emotional consequences of phubbing, such as feelings of sadness and being ignored [2, 10, 16, 22, 28]. The results are also in line with prior studies demonstrating the substantial influence of partner behaviors on women’s mental health during the perinatal period, including behaviors related to the use of new media [27].

Particularly noteworthy are the findings reported by Sobol et al. [27] in a Polish sample, which—similarly to the present study—revealed a significant association between problematic use of new media by the partner during the third trimester of pregnancy and increased depressive symptoms in women, alongside the absence of such an association in the first trimester. This pattern suggests that the third trimester may represent a particularly sensitive period during which women respond more strongly to partner behaviors and emotional availability. This result may be interpreted within the framework of the “nesting” concept, according to which women prepare their environment for the arrival of the child [18]. During this time, the partner’s support and presence become especially important for the woman’s subjective sense of safety for the child. A partner who exhibits emotional neglect or indifference through phubbing may evoke feelings of threat, anxiety, irritation, and loneliness in the woman as childbirth approaches. These findings may also be understood in light of evidence indicating that, in late pregnancy, many women experience reduced perceived attractiveness due to physical changes in their bodies [21]. Consequently, women during this period may be particularly sensitive to certain partner behaviors—such as problematic mobile phone use—which may be interpreted as signals of disinterest or even a shift of attention toward other women (cf. [7, 30]).

In the context of the Interpersonal Process Model of Intimacy by Reis and Shaver [23], the obtained results can be interpreted as indicating that partner phubbing may significantly disrupt the process of intimacy development within the relationship, thereby increasing the severity of depressive symptoms. When a partner directs attention to a phone rather than to interpersonal communication, the sequence of personal disclosure and the partner’s responsive reaction—perceived as attentive, understanding, and supportive—is interrupted. This disruption occurs already at the stage of disclosure, as women may limit both the frequency and depth of sharing their experiences. At the same time, phubbing weakens the perception of partner responsiveness, as such behavior may be interpreted as a lack of engagement, inattention, or devaluation of the communicated content. Consequently, the partner’s responses are not perceived as validating, which hinders the development of a sense of being understood and supported. These disruptions may lead to a breakdown of the positive, reinforcing dynamics of intimacy, in which disclosure fosters responsiveness, which in turn deepens closeness and encourages further openness. Instead, a negative spiral may emerge, involving emotional withdrawal, decreased trust, and reduced relationship satisfaction (see [23]. It is important to emphasize that in the third trimester of pregnancy, the significance of these mechanisms may be particularly pronounced due to the increased need for emotional support, a sense of security, and partner involvement in the face of the approaching childbirth. During this period, even subtle signals of inattention may be perceived as particularly distressing, making phubbing disproportionately disruptive to intimacy processes and emotional regulation within the relationship.

Interestingly, no association was found between phubbing as self-reported by men and the severity of women’s postpartum depressive symptoms. These results can be interpreted to suggest that men are sometimes unaware that their use of a mobile phone in the presence of their partner may be hurtful, as it can be perceived as a sign of disrespect or interest in someone else [25]. Moreover, the way a woman perceives and interprets her partner’s behavior is influenced by many factors, such as her level of trust in him, the quality of their relationship, their prior relational experiences, and the woman’s mood [11]. This result is consistent with several previous studies. For example, Carnelley et al. [6] demonstrated that daily perceived phubbing was significantly associated with poorer relationship quality, whereas phubbing measured using traditional self-report scales showed no such association. Similarly, findings reported by Frackowiak et al. [10] suggest that the harmful effects of phubbing are largely determined by the subjective perceptions of the individual experiencing the behavior.

The findings of the present study carry important practical implications. They indicate the need for psychoeducational interventions for couples that address the negative effects of phubbing, particularly in the later stages of pregnancy. Moreover, the results suggest that men may be unaware of how their mobile phone use is perceived by their partners. For this reason, it is especially important to raise awareness among expectant fathers during pregnancy regarding the significance of demonstrating attention and respect toward their partners in everyday interpersonal communication.

Despite several notable strengths—such as the pioneering nature of the study in examining the impact of phubbing during pregnancy on postpartum depressive symptoms, the use of a longitudinal research design, and the inclusion of both actor and observer perspectives in the assessment of phubbing—the study is also subject to certain limitations. Most notably, methodological constraints should be acknowledged. A key limitation of the present study is the relatively small sample size, which resulted from practical constraints that limited our ability to recruit a larger group of participants. This issue has important methodological implications, particularly with regard to statistical power. A small sample reduces the likelihood of detecting true effects, thereby increasing the risk of Type II errors. At the same time, limited sample size may also elevate the risk of Type I errors, as statistically significant findings in small samples can sometimes reflect random variation rather than true underlying effects [19]. Moreover, parameter estimates obtained from small samples—especially in more complex models such as cross-lagged panel models—may be less stable and more sensitive to sampling fluctuations. Consequently, the findings should be interpreted with caution, as some associations may have gone undetected, while others may not be robust. Future research based on larger samples is needed to replicate and extend these results, as well as to provide more reliable estimates of the observed relationships. Another limitation of the study is that we did not control for several important variables, such as relationship satisfaction, perceived social support, and stressful experiences. Moreover, a certain inconsistency in the measurement of phubbing should be mentioned. Phubbing among men was assessed using a standardized self-report scale, whereas women’s perceived phubbing was measured with a brief two-item daily diary. This discrepancy may be one possible explanation for the lack of association between men’s self-reported behavior and depressive symptoms in women.

In conclusion, the main finding of this study is that perceived partner phubbing during the third trimester of pregnancy is prospectively associated with more severe postpartum depressive symptoms in women. These results highlight the need to increase expectant fathers’ awareness of the potential negative consequences of phubbing, as well as of women’s heightened sensitivity to partner behaviors related to new media use in late pregnancy. The findings may inform the development of couple-based interventions focusing on psychoeducation about phubbing and its effects, along with communication skills training. However, further research is needed to replicate and validate these results. Future studies should consider using additional, including observational, measures of phubbing and examine potential moderators of the relationship between perceived phubbing and depressive symptoms.

## Supplementary Information

Supplementary Material 1.

## Data Availability

Data were deposited into the Open Science Framework https://osf.io/xfszc
