# Supplementary material for: Phubbing in pregnancy as a predictor of postpartum depressive symptoms: preliminary results
Source: BMC Psychol. 2026 Jul 7;14:1410. doi: 10.1186/s40359-026-04991-8 (PMC13625377; doi:10.1186/s40359-026-04991-8)
Supplement: Supplementary file 1 [file 40359_2026_4991_MOESM1_ESM.docx]

**Supplementary materials**

**Table S 1**

Correlations amongst medical and sociodemographic variables and women’s depressive symptoms.

|  | EPDS I | EPDS III | EPDS PP |
| --- | --- | --- | --- |
| 1. The presence of a chronic disease^ | -.09 | .18 | .31 |
| 2. Marital status^^ | .24 | .09 | -.05 |

*Note.* EPDS = Edinburgh Postnatal Depression Scale; I = measurement in the first trimester; III = measurement in the third trimester; PP = measurement after delivery; ^ 1 = no, 2 = yes; ^^ 1 = partnered, 2 = married

**Table S 2**

Results of cross-lagged analysis.

| Path | Dependent | Predictor | SPC | Z | P |
| --- | --- | --- | --- | --- | --- |
| Autoregressive | EPDS PP | EPDS III | 0.28 | 1.89 | 0.059 |
|  | phubbing PP | phubbing III | 0.60 | 3.50 | <0.001 |
| Cross-lagged | EPDS PP | phubbing III | 0.54 | 3.60 | <0.001 |
|  | phubbing PP | EPDS III | 0.02 | 0.13 | 0.897 |
| Correlations | EPDS III | phubbing III | 0.39 | 1.91 | 0.056 |
|  | EPDS PP | phubbing PP | 0.04 | 0.18 | 0.858 |
|  |  |  |  |  |  |

*Note.* SPC = standardized path coefficient; SE = standard error; 95% Cis = bootstrap generated 95% confidence intervals for SPC; EPDS = Edinburgh Postpartum Depression Scale; III = measurement in the third trimester; PP = measurement
